# *DICER-LIKE2* Plays a Primary Role in Transitive Silencing of Transgenes in *Arabidopsis*

**DOI:** 10.1371/journal.pone.0001755

**Published:** 2008-03-12

**Authors:** Sizolwenkosi Mlotshwa, Gail J. Pruss, Angela Peragine, Matthew W. Endres, Junjie Li, Xuemei Chen, R. Scott Poethig, Lewis H. Bowman, Vicki Vance

**Affiliations:** 1 Department of Biological Sciences, University of South Carolina, Columbia, South Carolina, United States of America; 2 Department of Biology, University of Pennsylvania, Philadelphia, Pennsylvania, United States of America; 3 Department of Botany and Plant Sciences and Institute of Integrative Genome Biology, University of California Riverside, Riverside, California, United States of America; University of Leeds, United Kingdom

## Abstract

Dicer-like (DCL) enzymes play a pivotal role in RNA silencing in plants, processing the long double-stranded RNA (dsRNA) that triggers silencing into the primary short interfering RNAs (siRNAs) that mediate it. The siRNA population can be augmented and silencing amplified via transitivity, an RNA-dependent RNA polymerase (RDR)-dependent pathway that uses the target RNA as substrate to generate secondary siRNAs. Here we report that *Arabidopsis DCL2*–but not *DCL4*-is required for transitivity in cell-autonomous, post-transcriptional silencing of transgenes. An insertion mutation in *DCL2* blocked sense transgene-induced silencing and eliminated accumulation of the associated *RDR*-dependent siRNAs. In hairpin transgene-induced silencing, the *dcl2* mutation likewise eliminated accumulation of secondary siRNAs and blocked transitive silencing, but did not block silencing mediated by primary siRNAs. Strikingly, in all cases, the *dcl2* mutation eliminated accumulation of all secondary siRNAs, including those generated by other DCL enzymes. In contrast, mutations in *DCL4* promoted a dramatic shift to transitive silencing in the case of the hairpin transgene and enhanced silencing induced by the sense transgene. Suppression of hairpin and sense transgene silencing by the P1/HC-Pro and P38 viral suppressors was associated with elimination of secondary siRNA accumulation, but the suppressors did not block processing of the stem of the hairpin transcript into primary siRNAs. Thus, these viral suppressors resemble the *dcl2* mutation in their effects on siRNA biogenesis. We conclude that *DCL2* plays an essential, as opposed to redundant, role in transitive silencing of transgenes and may play a more important role in silencing of viruses than currently thought.

## Introduction

RNA silencing is an ancient network of highly related pathways that repress gene expression in eukaryotic organisms by means of small regulatory RNAs [1]–[5]. The mechanism is triggered by dsRNA and specifically targets any related RNA (post-transcriptional pathways) or DNA (transcriptional gene silencing). Key steps in the process are: 1) the dsRNA trigger is cut into 21–24 nucleotide (nt) short-interfering (si) RNA duplexes by a ribonuclease III-like enzyme termed Dicer, 2) one strand of the siRNA duplex associates with an argonaute-like protein to form the core of a silencing effector complex, and 3) the siRNA directs the complex to complementary genetic elements. In post-transcriptional gene silencing, the complex targets mRNA, which is then cleaved by the ribonuclease-H activity of argonaute (AGO).

Higher plants, *C. elegans*, and fungi additionally amplify silencing via transitivity, a pathway that produces dsRNA and additional siRNAs from the targeted mRNA [6], [7]. Production of dsRNA in transitive silencing depends on cellular RNA-dependent RNA polymerase (RDR) activity, and the siRNAs generated are termed secondary siRNAs, while those that derive from the initial dsRNA trigger are termed primary siRNAs. In plants, production of both primary and secondary siRNAs entails cleavage of dsRNA by the Dicer-like (DCL) family of enzymes [6], [8]. In plants, therefore, transitivity not only increases the siRNA population, but also degrades the target mRNA in the process of making secondary siRNAs, thereby amplifying silencing in two ways. Interestingly, secondary siRNA production itself is not a mechanism of target degradation in *C. elegans* because antisense secondary siRNAs are transcribed directly from the target mRNA template in that organism [9], [10]. The importance of transitive silencing has recently come to light in studies in *C. elegans* showing that the vast majority of siRNAs that accumulate during RNA silencing in that organism are secondary siRNAs [9]. Transitive silencing is also important in plants, where it is essential for some types of transgene-induced silencing [11] and thought to play a key role in defense against viruses [12], [13].

Although RNA silencing was initially considered simply a novel defense mechanism against viruses and other invading nucleic acids, the subsequent discovery of endogenous small regulatory RNAs led to the realization that it is a fundamental genetic regulatory mechanism in eukaryotic organisms. There are three major classes of endogenous small regulatory RNAs in plants: micro (mi) RNA, trans-acting (ta) siRNA, and heterochromatin-associated (hc) siRNA [14]–[18]. The biogenesis and mechanism of action of the different silencing-associated small regulatory RNAs are highly related; however, hc-siRNAs suppress transcription as opposed to mediating RNA degradation and, in animal systems, miRNAs primarily repress translation. Many of the enzymes involved in RNA silencing are encoded by multigene families, allowing for the possibility of diverse, specialized pathways [4], [19]. In *Arabidopsis*, there are four *DCL*, ten *AGO*, and six *RDR* genes. The roles of the four *Arabidopsis DCL* genes in small RNA biogenesis and silencing have been the subject of intense study, and the picture that has emerged is one of primary roles for many of the enzymes, plus functional redundancy. Thus, *DCL1* is required for the biogenesis of 21-nt miRNAs, and *DCL3* is responsible for the biogenesis of 24-nt hc-siRNAs [14], [15], [20]. *DCL4* seems the most versatile, being required for production of 21-nt ta-siRNAs, which derive from *RDR6*-generated dsRNA, as well as for production of 21-nt *RDR6*-independent siRNAs from hairpin transgenes, in which case dsRNA can be produced directly by transcription of an engineered inverted repeat [21]–[25]. *DCL4* also plays a primary role in production of siRNAs from viruses, with *DCL2* as the substitute if *DCL4* has been inactivated [25]–[28]. Although *DCL2* has been reported to produce an endogenous siRNA from a convergently transcribed and overlapping gene pair [29], it is otherwise considered to play a subordinate and redundant role in siRNA biogenesis. Another possibility, however, is that the primary role of *DCL2* has simply not yet been identified.

With this idea in mind, we undertook a systematic study of the impact of mutations in *DCL* genes and the effect of viral silencing suppressors on siRNA biogenesis in *Arabidopsis thaliana* carrying different types of silenced transgene. Our experiments focused on three general cases: 1) sense transgene-induced silencing, in which case a construct that was designed to express a reporter gene becomes post-transcriptionally silenced instead, 2) hairpin transgene-induced silencing of a sense transgene, in which case a transgene that produces a self-complementary transcript is used to target an expressing, homologous sense transgene, and 3) processing of the hairpin transcript itself (hairpin transgene self-silencing). We expected that these cases might differ in their *DCL* requirements because sense transgene-induced silencing requires a set of genes that are not required for hairpin-induced silencing of transgenes [30], [31]. Some of these genes are thought to mediate production of dsRNA from the sense transgene transcript and include *RDR6*, *AGO1,* and *SGS3*–which encodes a coiled-coiled domain protein of unknown function, while *HEN1* encodes a methylase that acts downstream of dsRNA and stabilizes siRNAs [32].

The transgenes we used differ in several important respects from those in other studies. First, no other study to date has analyzed the *DCL* requirements for sense transgene-induced silencing. The exact nature of the silencing trigger is still unknown in this type of silencing, but it is thought that the locus gives rise to an aberrant transcript, which might lack normal 5′ or 3′ ends. The aberrant transcript becomes a template for RDR6, which synthesizes the complementary strand and produces dsRNA. Second, with respect to hairpin transgene-induced silencing, the loop portion of the hairpin construct we used is retained in the mature transcript and has sequence identical to that of the targeted sense transgene. Thus, this hairpin construct is unusual in that it has a region that requires *RDR6* to produce dsRNA in addition to one that can directly produce dsRNA, and siRNAs from both regions can target the sense transgene. Furthermore, although hairpin transgene-induced silencing has been the subject of numerous studies, no other study has examined the fate of the hairpin transcript itself and the *DCL* requirements for self-silencing of the hairpin. Lastly, we used transgenes driven by the ubiquitously expressed cauliflower mosaic virus (CaMV) 35S promoter. Consequently, silencing occurs throughout the plant without the need for a silencing signal.

In all three cases of silencing we examined, accumulation of secondary siRNAs required *DCL2* but not *DCL4*, providing evidence that DCL2 plays an essential, as opposed to redundant, role in transitive silencing of transgenes. This result strongly suggests that there are natural targets of silencing for which DCL2 plays a primary role. Furthermore, we found that viral suppressors of silencing and a *dcl2* null mutation had similar effects on accumulation of secondary siRNAs, supporting a primary role for *DCL2* in antiviral defense.

## Results

### *DCL2* but not *DCL4* is Required for Sense Transgene-induced Silencing

**Figure 1:**
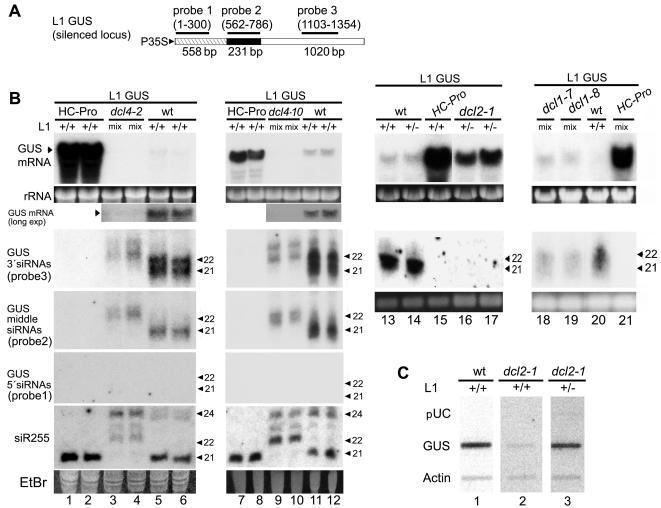
Sense Transgene-induced Silencing is Impaired in *dcl2* Mutant Plants, but Enhanced in *dcl4* Mutants. (A) The diagram shows the coding region of the silenced GUS sense transgene in line L1, plus the coordinates and positions of the probes used throughout this work for detecting GUS mRNA and siRNA. Antisense polarity probes will be indicated by an asterisk. The position and length in base pairs (bp) of the stem, deletion, and loop regions of the ΔGUS-SUG hairpin construct (Figures 2–3) are also shown. (B) Accumulation of GUS mRNA, GUS siRNAs, and siR255 in wild type (wt) and mutant plants carrying the L1 GUS locus was determined using RNA gel blot analysis. ^32^P-labelled RNA probes were used for hybridization except that a DNA oligonucleotide probe was used for the siR255 blots and a full length GUS cDNA probe was used for the high molecular weight (HMW) RNA blot of lanes 18–21. Otherwise, probe 3*, which has antisense polarity, was used to detect GUS mRNA. Probes for GUS siRNA all had sense polarity and, therefore, detected the antisense strand. The positions of 21- and 22-nt RNA size markers (Ambion Decade™ Marker system) are indicated on the right of the low molecular weight (LMW) RNA blots. Grouped lanes are all from the same gel, blot, and exposure. A longer exposure of the GUS mRNA band in lanes 3–6 and 9–12 is shown directly below the rRNA band. LMW RNA blots were successively stripped and hybridized with the indicated probes. Genotypes and the zygosity of the L1 locus are indicated at the top of the lanes. The designation “mix” indicates that a segregating F2 population that was a mix (theoretically about 2:1) of L1 GUS hemizygotes and homozygotes was used for RNA isolation; +/− and +/+ indicate hemizygous and homozygous for L1 GUS, respectively. Ethidium bromide (EtBr) stained rRNA and the major RNA species in LMW RNA are shown as loading controls. (C) Relative levels of transcription of the GUS transgene in plants wild type for *DCL2* (lane 1) and in *dcl2-1* mutant plants homozygous (lane 2) or hemizygous (lane 3) for L1 GUS were determined in isolated nuclei. Nuclear transcripts from these plants were labeled with ^32^P by run-off transcription and then hybridized to slot blots loaded with plasmid DNA containing GUS, actin, and pUC19 empty vector sequence.

To study sense transgene-induced silencing, we used the L1 transgenic line, which carries a direct repeat of the T-DNA insert and is silenced for the *uidA* gene encoding β-glucuronidase (GUS) [33]. Silencing of the GUS transgene in line L1 initiates at about the time the plant bolts and is characterized by very low accumulation of GUS mRNA and concomitant accumulation of GUS siRNAs that are *RdR6*-dependent [31] and 21- and 22-nucleotides (nt) in length (Figure 1A and 1B, lanes 5–6 and 11–12; [31]). These siRNA size classes have previously been attributed to the activity of DCL4 and DCL2, respectively [21]–[24]. About equal amounts of the two size classes are detected using a probe to the 3′-most region of the transcript, whereas only 21-nt siRNAs are detected with a probe to the central region, and no siRNAs of any size class can be detected using a probe to the 5′-most region (Figure 1B, lanes 5–6 and 11–12). This drop-off in accumulation of siRNAs at the 5′ end of the L1 GUS transcript was not seen in an earlier study [31], possibly because the 5′ probe used by that group extended 258-nt further 3′ than ours.

To identify which *DCL* genes are required for sense transgene-induced silencing, we crossed the L1 line with *dcl* mutant lines and examined the accumulation of GUS mRNA and siRNAs in F2 progeny that were homozygous for the *dcl* mutation and also carried at least one copy of the L1 GUS transgene. The post-transcriptional silencing induced by a single copy of the L1 GUS locus is comparable to that observed in plants homozygous for the locus (Figure 1B, compare lanes 13 and 14). An L1 GUS line that expressed the P1/HC-Pro viral suppressor of silencing was used as a positive control showing expression of the GUS transgene. The *dcl2* and *dcl3* mutant lines were null mutants generated by T-DNA insertion, whereas the *dcl1* mutant lines were partial loss of function mutants. The *dcl4* mutant lines had point mutations in *DCL4* that eliminated or greatly reduced accumulation of the 21 nt species of ta-siR255 (Figure 1B, lanes 3–4 and 9–10; [23]). Because 21 nt ta-siRNAs are produced by DCL4 [21]–[23], this result confirms that these *dcl4* mutant lines are highly deficient for DCL4 activity. All F2 progeny homozygous for the *dcl3* mutation were transcriptionally silenced for GUS, whether they carried one or two copies of the GUS transgene (Mlotshwa and Vance, manuscript in preparation), preventing examination of the role of *DCL3* in sense transgene-induced silencing in this system. Transcriptional silencing could be avoided, however, in the *dcl2* mutant by using progeny that were hemizygous for the GUS transgene: Run-off transcription assays showed that whereas *dcl2* mutant progeny homozygous for the L1 locus were transcriptionally silenced for GUS, ones hemizygous for the L1 locus were not (Figure 1C). The transcriptional silencing induced by T-DNA insertion mutations is likely due to the CaMV 35S promoter in the engineered T-DNA [34], [35]. This propensity of the L1 locus to become transcriptionally silenced in the presence of additional copies of the CaMV promoter highlights the importance of examining siRNA accumulation before concluding that absence of GUS mRNA or activity indicates that the locus is post-transcriptionally silenced. Transcriptional silencing was not a problem in the *dcl1* or *dcl4* mutant lines, which were not T-DNA insertion mutants.

Silencing of the L1 GUS transgene was severely impaired in *dcl2* mutant plants, as shown by greatly increased accumulation of GUS mRNA and the absence of GUS siRNAs (Figure 1B, compare lanes 14, 16, and 17). Thus, *DCL2* plays a primary role in this *RDR6*-dependent silencing pathway. In contrast, silencing of the L1 GUS transgene was enhanced in both *dcl4-2* and *dcl4-10* mutant plants, as shown by reduced accumulation of GUS mRNA compared to wild type plants (Figure 1B, lanes 3–6 and 9–12). Thus, although DCL4 activity produced the major fraction of GUS siRNAs that accumulated in the wild type background, neither *DCL4* nor *DCL4*-dependent 21-nt siRNA is required for sense transgene-induced silencing. The enhancement of silencing seen in the *dcl4* mutants might indicate some inhibitory role of *DCL4* or an earlier onset of silencing due to the accelerated juvenile-to-adult transition seen in these mutants [22], [23]. The *dcl1* mutant plants were slightly impaired for silencing of L1 GUS (Figure 1B, lanes 18–20), suggesting that *DCL1* plays a facilitating role in sense transgene-induced silencing like that reported for hairpin transgene-induced silencing of an endogenous gene [36].

Although accumulation of GUS mRNA in the *dcl2* mutant plants is much greater than in wild type, it is considerably less than seen in plants expressing P1/HC-Pro (Figure 1B, lanes 14–17). Part of this differential is likely a gene dosage effect due to the fact that the P1/HC-Pro line is homozygous for L1 GUS, whereas the *dcl2* mutant is hemizygous for the transgene. The remaining differential might indicate that absence of DCL2 does not completely eliminate sense transgene-induced silencing, but only reduces it. Finally, the absence of GUS siRNAs of all size classes in the *dcl2* mutant plants suggests that DCL2 or the 22-nt class of siRNAs it generates is necessary for DCL4 and other DCL enzymes to be active on this type of substrate.

### *DCL2* but not *DCL4* is Required for Transitivity in Self-silencing of a Hairpin Transgene

**Figure 2:**
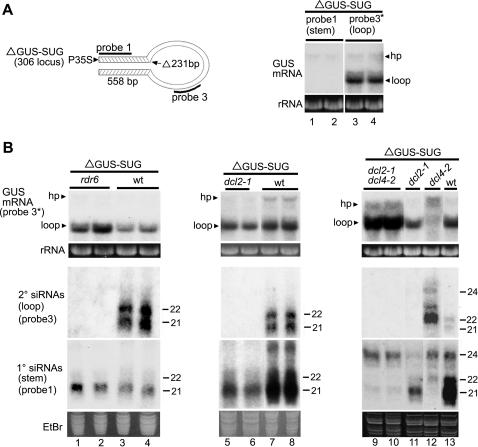
*DCL2* and *DCL4* have Distinct Roles in Self-silencing of the ΔGUS-SUG Hairpin Transgene. (A) The diagram shows the coding region of the ΔGUS-SUG hairpin (hp) transgene drawn to illustrate the double-stranded configuration of the transcript. The locations of the hybridization probes and the 231-bp deletion are indicated. Probes 1 and 3 are as specified in Figure 1A. Accumulation of GUS mRNA in the ΔGUS-SUG line 306-1 was determined using RNA gel blot analysis and RNA probes. Duplicate samples of two different RNA preparations were run on one gel and blotted onto one membrane, after which the membrane was cut in half. One half was hybridized with probe 1 and the other with probe 3*; probe 1* gave the same result as probe 1 (data not shown). Ethidium bromide stained rRNA is shown as a loading control. (B) Accumulation of GUS mRNA and siRNAs in wild type (wt) and mutant lines carrying the ΔGUS-SUG transgene of line 306-1 was determined using RNA gel blot analysis with RNA probes. Probe 3* was used to detect GUS mRNA, while probes for GUS siRNAs all had sense polarity. Grouped lanes are all from the same gel, blot, and exposure. LMW RNA blots were successively stripped and hybridized with the indicated probes. The positions of 21- to 24-nt RNA size markers are indicated. Ethidium bromide stained rRNA and the major RNA species in LMW RNA are shown as loading controls.

**Figure 3:**
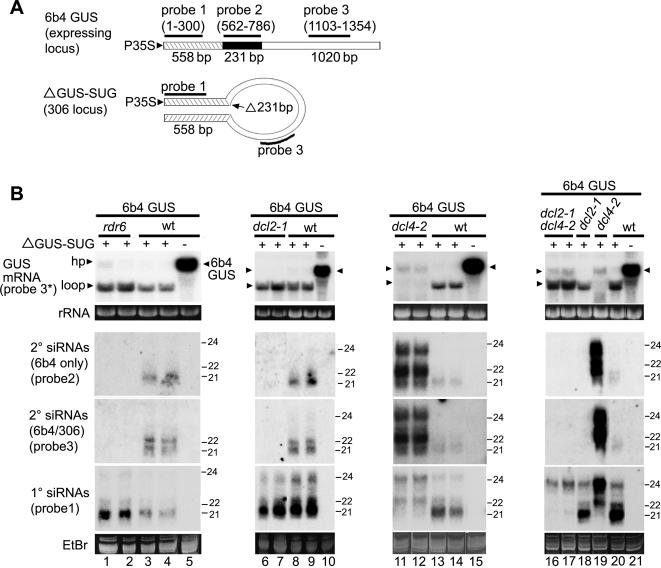
Secondary siRNAs in Hairpin-induced Silencing are Eliminated by *dcl2* but greatly Increased by *dcl4* Mutations. (A) Diagrams of the coding regions of the 6b4 GUS expressing locus and the ΔGUS-SUG hairpin show the regions and probes first described in Figures 1 and 2. The solid bar in the 6b4 GUS diagram corresponds to the 231-bp deletion in the ΔGUS-SUG hairpin construct. (B) Accumulation of GUS mRNA and siRNAs in wt and mutant lines transgenic for both 6b4 GUS and the ΔGUS-SUG hairpin was determined using RNA gel blot analysis with RNA probes. Line 6b4, which carries only the 6b4 GUS transgene, was used as a control. Probe 3* was used to detect GUS mRNA, while probes for GUS siRNAs all had sense polarity. Grouped lanes are all from the same gel, blot, and exposure. LMW RNA blots were successively stripped and hybridized with the indicated probes. The positions of 21- to 24-nt RNA size markers are indicated. Ethidium bromide stained rRNA and the major RNA species in LMW RNA are shown as loading controls.

Our initial studies of hairpin transgene-induced silencing used northern analysis to examine the fate of the hairpin transcript itself. The self-complementary ΔGUS-SUG transgene in line 306-1 [30] consists of the GUS coding sequence with a 231-nt deletion after nucleotide 558 and an inverted duplication of the 5′ proximal 558-nt at the 3′ end (Figure 2A and 3A; [37]). The 558-nt self-complementary regions form the stem of the hairpin, while the intervening GUS sequence forms the loop. Unlike hairpin transgene constructs that have an intron separating the self-complementary regions, the loop portion of the ΔGUS-SUG transgene is not eliminated from the mature transcript. As a result, it is possible to examine siRNA biogenesis not only from the stem of the hairpin, which can pair to form dsRNA, but also from the loop, which requires the action of an RDR to generate dsRNA.

Not surprisingly for a transgene that is a strong inducer of silencing, very little full-length ΔGUS-SUG transcript accumulated in line 306-1 (Figure 2A, lanes 1–4, band labeled hp). Unexpectedly, however, significant quantities of a smaller RNA species accumulated. This species could be detected with a probe specific for the loop (Figure 2A, lanes 3–4) but not with one specific for the stem (Figure 2A, lanes 1–2), showing that it corresponded to the loop portion of the hairpin transcript. Its size, as determined by migration in agarose gels, corresponds to that of the entire loop (data not shown). The presence of GUS siRNAs confirms that the ΔGUS-SUG transgene in line 306-1 is silenced (Figure 2B, lanes 3–4). Preferential accumulation of the loop mRNA, however, indicates that degradation of the hairpin transcript occurs mainly via processing of the stem of the hairpin. Degradation of the loop portion of the transcript, which requires transitive silencing, apparently does not occur very effectively in this system. Analysis of ΔGUS-SUG mRNA and siRNA accumulation in an *rdr6* mutant backgound confirms that elimination of the full-length ΔGUS-SUG transcript and accumulation of siRNAs from the stem of the hairpin are *RDR6*-independent, whereas accumulation of siRNAs from the loop is *RDR6*-dependent: Loop mRNA, but little full-length ΔGUS-SUG transcript, accumulates in *rdr6* mutant plants, as in wild type; however, siRNAs from the loop are eliminated by the *rdr6* mutation, while those from the stem are largely unaffected (Figure 2B, lanes 1–2). Thus, as expected, siRNAs from the stem are primary siRNAs, while those from the loop are secondary siRNAs and part of the transitive silencing pathway. The increased accumulation of GUS loop mRNA in the *rdr6* mutant compared to wild type provides additional confirmation that degradation of the loop occurs via transitive silencing.

The ΔGUS-SUG primary siRNAs are predominantly 21-nt (Figure 2B, lanes 1–4), suggesting that DCL4 is normally responsible for processing the dsRNA stem of the hairpin. In contrast, secondary siRNAs from the hairpin transcript are approximately equal parts 21- and 22-nt (Figure 2B, lanes 3–4), as seen for the 3′ end of the L1 GUS sense transgene. To examine the roles of *DCL2* and *DCL4* in self-silencing of the ΔGUS-SUG transcript, we crossed line 306-1 with *dcl2* and *dcl4* mutant lines and examined F2 progeny homozygous for the *dcl* mutation. The hairpin transgene remains silenced in both *dcl* mutants, as indicated by the presence of GUS siRNAs and little accumulation of full-length ΔGUS-SUG mRNA (Figure 2B, lanes 11–13). In the *dcl4* mutant, however, accumulation of loop mRNA is nearly eliminated (Figure 2B, lanes 12–13), suggesting that impairing DCL4 activity promotes transitive silencing of the hairpin transcript. Enhanced accumulation of loop siRNAs and elimination of the majority of primary siRNAs in the *dcl4* mutant compared to wild type (Figure 2B, lanes 12–13) are consistent with a shift to transitive silencing in the mutant. Increased accumulation of full-length ΔGUS-SUG mRNA in the *dcl4* mutant compared to wild type (Figure 2B, lanes 12–13) indicates that the reduction in loop mRNA accumulation in the mutant is not due to increased non-specific degradation. Furthermore, it suggests that transitive silencing of the hairpin transcript is not as effective as the *DCL4*-dependent pathway that normally eliminates the stem.

In contrast to the shift to transitive silencing produced by the *dcl4* mutation, the *dcl2* mutation impairs transitivity: No loop siRNAs accumulate in the *dcl2* mutant (Figure 2, lanes 5–8). Because loop siRNAs are RDR6-dependent secondary siRNAs (Figure 2B, lanes 1–4), this result implies that *DCL2* is required for transitive self-silencing of the hairpin transgene. In addition, accumulation of stem siRNAs, which are predominantly 21-nt, is reduced in the *dcl2* mutant (Figure 2B, lanes 5–8), suggesting that DCL2 facilitates DCL4 production of primary siRNAs or enhances their stability. The *dcl2* mutation blocks transitive silencing even in a *dcl4* mutant background: Loop siRNAs are absent and high levels of loop mRNA accumulate in *dcl2 dcl4* double mutant plants (Figure 2B, lanes 9–10), indicating that degradation of the hairpin transcript occurs mainly via processing of the dsRNA stem of the hairpin in the absence of both *DCL2* and *DCL4*. Thus, the shift to transitive silencing observed in *dcl4* mutant plants requires *DCL2*. Processing of the dsRNA stem of the hairpin transcript in the *dcl2 dcl4* double mutant presumably involves DCL3, DCL1 or residual low-level DCL4 activity, as 24-nt and a small amount of 21-nt primary (stem) siRNAs accumulate in these plants (Figure 2B, lanes 9–10).

Altogether, these results show that *DCL2* is required for production of *RDR6*-dependent siRNAs from a hairpin transgene as well as from a sense transgene. In addition, self-silencing of the hairpin transcript provides another example of the hierarchical action of the DCL proteins and reveals that different DCL proteins are preferentially associated with different mechanisms of transcript degradation. In wild type plants, the major pathway for degradation of the ΔGUS-SUG hairpin transcript appears to be *DCL4*-dependent processing of the stem into primary siRNAs. In a *dcl4* mutant, transitive silencing is prominent and involves *DCL2*- and *RDR6*-dependent production of secondary siRNAs. When both *DCL2* and *DCL4* are defective, degradation again occurs via processing of the stem into primary siRNAs, but this time involves *DCL3* and possibly *DCL1*.

### *DCL2* but not *DCL4* is required for transitivity in hairpin transgene-induced silencing of a sense transgene

To analyze hairpin transgene-induced silencing of a sense transgene, we used the ΔGUS-SUG locus in line 306-1 to target an expressing GUS locus (Figure 3A). Line 6b4 carries a GUS sense transgene that is not silenced [30], and 6b4 plants accumulate high levels of GUS mRNA but no GUS siRNAs (Figure 3B, lane 5). However, the GUS-expressing locus in line 6b4 is silenced in the presence of the ΔGUS-SUG construct, as evidenced by the loss of GUS activity [30]. For our experiments, therefore, we used a 6b4/306 transgenic line obtained by crossing the 6b4 and 306-1 lines. The full-length GUS and ΔGUS-SUG transcripts are similar in size, but the ΔGUS-SUG sequence is 327-nt longer (Figure 3A).

Northern analysis shows that the 6b4/306 line is silenced for both transgenes because little or no full-length transcript from either one accumulates, but loop mRNA from the hairpin transcript and GUS siRNAs do (Figure 3B, lanes 3–4). To detect GUS siRNAs in these experiments, we used the same stem and loop probes (1 and 3, respectively) as in the previous section, but also included probe 2, which corresponds to the region deleted in the ΔGUS-SUG construct (Figure 3A). In line 6b4/306, probes 1 (stem) and 3 (loop) can detect siRNAs arising from either the ΔGUS-SUG transgene or the GUS sense transgene, while probe 2 is specific to the GUS sense transgene. Accumulation of the GUS siRNAs detected by probes 2 and 3 in 6b4/306 plants was eliminated in the *rdr6* mutant background (Figure 3B, lanes 1–2), confirming that these are *RDR6*-dependent secondary siRNAs and part of the transitive silencing pathway. Accumulation of the GUS siRNAs detected by probe 1, however, was not reduced by the *rdr6* mutation (Figure 3B, lanes 1–4), showing that even in the presence of the GUS sense transgene, these are mostly primary siRNAs from the stem of the hairpin transcript. This observation plus the accumulation of loop mRNA suggests that hairpin transgene-induced silencing of the GUS-sense transgene in line 6b4/306 does not have a large transitive component. Indeed, silencing of the GUS-sense transgene in line 6b4/306 is largely unaffected by the *rdr6* mutation, as little or no full-length GUS mRNA accumulates in the *rdr6* mutant plants (Figure 3B, lanes 1–2), consistent with earlier work showing that hairpin transgene-induced silencing is *RDR6*-independent [30].

To examine the roles of *DCL2* and *DCL4* in ΔGUS-SUG-induced silencing of the GUS sense transgene, we performed crosses to make homozygous *dcl2*, *dcl4*, and *dcl2 dcl4* mutant progeny of line 6b4/306. The effects of the *dcl* mutations on silencing of the GUS sense transgene in these lines was very similar to their effects on self-silencing of the hairpin transgene shown above for lines carrying the hairpin locus alone (Figure 2B). In the 6b4/306 background, the *dcl4* mutation eliminated accumulation of loop mRNA and greatly increased accumulation of secondary siRNAs–including those (probe 2) that could be derived only from the GUS sense transgene transcript–providing additional evidence that impairing DCL4 activity promotes a shift to transitive silencing (Figure 3B, lanes 11–14, probes 2 and 3). The highly abundant secondary siRNAs in the *dcl4* mutant included 24-nt as well as 22-nt siRNAs, showing that both DCL3 and DCL2 produce secondary siRNAs from the targeted sense transgene when DCL4 is defective. The much greater increase in secondary siRNA accumulation caused by the *dcl4* mutation in lines carrying both transgenes than in those having the hairpin locus alone suggests that the GUS sense transgene transcript is a much better substrate for *RDR6*-dependent production of siRNAs than the hairpin transcript.

In contrast to *dcl4* enhancement of secondary siRNA accumulation, the *dcl2* mutation eliminated secondary siRNAs–including those (probe 2) that could be derived only from the GUS sense transgene mRNA (Figure 3B, lanes 6–9, probes 2 and 3). Accumulation of siRNAs detected by probe 1, which are mostly primary siRNAs, was not greatly affected (Figure 3B, lanes 6–9). The *dcl2* mutation also eliminated accumulation of secondary siRNAs in the *dcl4* mutant background (Figure 3B, compare lanes 16–19, probes 2 and 3) and restored accumulation of loop mRNA (Figure 3B, lanes 16–20). Thus, *DCL2* is required for transitive silencing of the sense transgene target of a hairpin transgene, as well as for transitive silencing of the hairpin itself.

The above results provide a basis for understanding the robust nature of hairpin transgene-induced post-transcriptional silencing. Whereas a sense transgene alone activates only an *RDR6*-dependent silencing pathway, a hairpin transgene activates multiple silencing pathways–including one(s) involving only *RDRP*-independent primary siRNAs. Consequently, in the presence of a homologous hairpin transgene, the sense transgene becomes a target for all the silencing pathways activated by the hairpin construct.

### The P1/HC-Pro and P38 viral suppressors block hairpin transgene-induced silencing of a sense transgene, but do not block processing of the stem of the hairpin transcript into primary siRNAs

Previous studies in our laboratory showed that the tobacco etch virus (TEV) P1/HC-Pro viral suppressor of silencing altered the accumulation of siRNAs in tobacco, eliminating those derived from sense transgenes and shifting the size distribution of ones derived from inverted repeat and amplicon transgenes [38], [39]. To determine the effect of P1/HC-Pro and other viral suppressors on siRNA biogenesis in the three cases of silencing examined above, we crossed the L1, 306-1, and 6b4/306 lines with *Arabidopsis* lines transgenic for P1/HC-Pro (from turnip mosaic virus) or P38 (from turnip crinkle virus). The P38 viral suppressor carried a C-terminal HA epitope tag [40], whereas P1/HC-Pro had no tag. In all cases, progeny carrying a viral suppressor transgene exhibited the developmental phenotype associated with expression of the suppressor in the parental line (data not shown).

**Figure 4:**
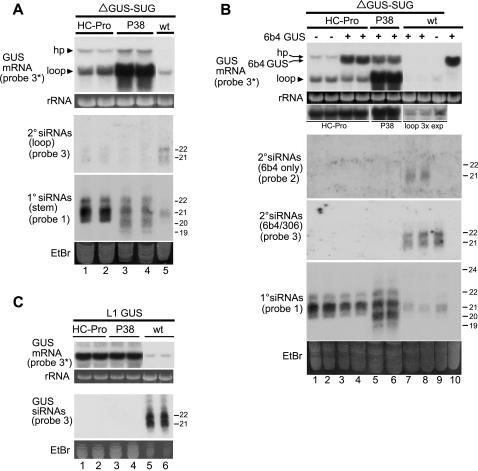
P1/HC-Pro and P38 Enhance ΔGUS-SUG Loop mRNA and Primary siRNA Accumulation, but Eliminate Secondary siRNAs. (A) Gel blot analysis of HMW and LMW RNA is shown for progeny of crosses between the ΔGUS-SUG hairpin line 306-1 and lines expressing the indicated viral suppressors of silencing. All procedures and designations are as described in the legend to Figure 2. Grouped lanes are all from the same gel, blot, and exposure. (B) RNA gel blot analysis is shown for lines that express the indicated viral suppressors and are transgenic for both the ΔGUS-SUG hairpin and 6b4 GUS expressing locus. Controls that carry 6b4 GUS alone (lane 10) or ΔGUS-SUG alone (lanes 1, 2, 9) are also included. All procedures and designations are as described in the legends to Figures 2 and 3. A longer exposure of the loop band in lanes 7–9 is also shown. Accumulation of viral suppressor mRNA in lanes 1–6 is shown for a duplicate gel that was blotted to one membrane, cut into sections corresponding to the indicated viral suppressors, and hybridized with an RNA probe specific for that suppressor; otherwise, grouped lanes are all from the same gel, blot, and exposure. (C) RNA gel blot analysis is shown for progeny of crosses between the silenced L1 GUS line and lines expressing the indicated viral suppressor. All procedures and designations are as described in the legend to Figure 1 except that only RNA probes were used. Grouped lanes are all from the same gel, blot, and exposure.

Both of these suppressors restored accumulation of GUS mRNA and eliminated accumulation of GUS siRNAs in progeny of crosses with the sense transgene silenced line L1 (Figure 4C), similar to the effect of TEV P1/HC-Pro on sense transgene-induced silencing in tobacco. Neither suppressor, however, enabled accumulation of high levels of the full-length ΔGUS-SUG transcript in progeny of crosses with the hairpin-transgenic line 306-1, although accumulation of the full-length transcript was increased (Figure 4A, lanes 1–5). In contrast to the full-length transcript, high levels of loop mRNA accumulated in both the P1/HC-Pro and P38 lines (Figure 4A, lanes 1–4). Thus, P1/HC-Pro and P38 have little or no inhibitory effect on processing the dsRNA stem of the hairpin, but they suppress the secondary siRNA-dependent pathway responsible for degradation of the loop. Consistent with this interpretation, P1/HC-Pro and P38 reduced accumulation of secondary (loop) siRNAs (Figure 4A, lanes 1–5). Interestingly, the suppressors also increased accumulation of primary (stem) siRNAs (Figure 4A, lanes 1–5). The relatively minor accumulation of full-length transcript in the P1/HC-Pro and P38 lines suggests that although most ΔGUS-SUG transcripts are degraded via processing of the dsRNA stem into primary siRNAs, some are degraded as a result of being targeted by primary siRNAs and that P1/HC-Pro and P38 block this latter pathway. The increase in primary siRNA accumulation in the viral suppressor lines compared to wild type plants is consistent with the idea that some full-length hairpin transcripts are degraded by transitive silencing in wild type plants, thereby reducing production of primary siRNAs. Alternatively, the suppressors might increase primary siRNA stability, perhaps by binding siRNA duplexes [41], [42].

In progeny of crosses with the 6b4/306 line, both P1/HC-Pro and P38 restored accumulation of full-length mRNA from the GUS sense transgene and eliminated secondary siRNAs (probes 2 and 3) (Figure 4B, lanes 3–8). In addition, both suppressors enhanced accumulation of loop mRNA and primary siRNAs. The enhancement of loop mRNA accumulation by P38 was so great that loop mRNA in wild type plants was barely detectable at the exposure appropriate for the samples from P38-expressing plants (Figure 4B, lanes 7–9), although it was clearly visible at a 3-fold longer exposure (Figure 4B, image of lanes 7–9 labeled “loop 3x exp”). These results are consistent with the effects of the suppressors on secondary siRNA-dependent silencing seen with the hairpin transgene alone (Figure 4A and 4B, lanes 1, 2, 9). Hybridizing a duplicate high molecular weight RNA blot with probes specific to the viral suppressors confirmed that both suppressors were being expressed (Figure 4B, panels in lanes 1–6 directly below rRNA).

The P38 viral suppressor produced several very interesting effects in the above experiments. P38 transgenic plants accumulated much higher levels of loop mRNA than P1/HC-Pro plants (Figure 4A, lanes 1–4 and 4B, lanes 3–6), suggesting that P38 is much more effective than P1/HC-Pro at preventing degradation of the loop. P38 also had a more pronounced effect than P1/HC-Pro on the size distribution of primary siRNAs, causing the accumulation of a closely spaced smaller species (Figure 4B, lanes 5–6), suggesting that altering the processing of small RNAs is part of the P38 mechanism of action.

Some of our viral suppressor results differ from those of another group. P1/HC-Pro inhibition of ta-siRNA accumulation [8] and P38 inhibition of DCL4 activity [28] is not evident in our lines (Figure 1B, siR255 probe; Figure 4A, B: primary siRNAs). Conversely, the enhancement of primary siRNA accumulation by P1/HC-Pro and P38 that we observe in hairpin transgene-induced silencing (Figure 4A, B) was not detected by the other group [8], [43]. We expect that differences in the plant lines as well as in the inducers and targets of silencing used by the two groups are likely responsible for such discrepancies. Consistent with this expectation and in agreement with our result, an independent group using our plant line observed that P1/HC-Pro does not block ta-siRNA accumulation [44]. It is also interesting to note that P1/HC-Pro enhancement of siRNA accumulation has previously been observed for a hairpin promoter sequence construct that induced transcriptional silencing in tobacco [45].

## Discussion

The present work demonstrates that *DCL2* is required for silencing induced by a sense transgene locus and for accumulation of secondary siRNAs in three mechanistically different examples of transgene silencing. Surprisingly, even *DCL4*-dependent 21-nt and *DCL3*-dependent 24-nt secondary siRNAs are eliminated by a *dcl2* null mutation, suggesting that DCL2 is required for DCL4 and DCL3 participation in production of *RdR6*-dependent siRNAs from these transgenes. Our results show that DCL2 plays a primary role in transitive silencing of transgenes and suggest that there are as-yet-unidentified natural substrates for which DCL2 is the primary DCL enzyme. One possible origin of natural substrates of DCL2 is foreign genes that are introduced into the plant genome during infection with pathogens like *Agrobacterium tumefaciens*. Such genes constitute a natural analogue of transgenes. In addition, our observation that a *dcl2* null mutation and two viral suppressors of silencing have the same effect on secondary siRNA accumulation suggests that natural substrates of DCL2 might be produced in viral infection. The similarity in effect of the *dcl2* mutation and the viral suppressors applies only to accumulation of secondary siRNAs, however, and not to suppression of silencing in general: The suppressors eliminated both hairpin and sense transgene-induced silencing, whereas the *dcl2* mutation impaired only the latter.

Although DCL2 was initially proposed to play an antiviral role [20], subsequent studies suggested that the enzyme functions in only a subordinate and redundant capacity in antiviral defense [25]–[28], [46]. A key piece of evidence for this conclusion is that 22-nt viral siRNAs accumulated primarily when DCL4 was inactive. Those studies, however, either involved only primary siRNAs or did not distinguish between primary and secondary siRNAs. In contrast, our results point to a requirement for *DCL2* specifically in the production of secondary siRNAs. *RDR6*-dependent production of secondary siRNAs is thought to be particularly important in slowing the systemic spread of viruses by allowing systemically invaded cells to respond before the virus starts replicating [12]. After replication is established, however, viral siRNA production no longer depends on *RDR6* [12], suggesting that any specific requirement for *DCL2* in antiviral defense might be transient and not detectable in bulk infected tissue.

The inhibition of transitivity by P1/HC-Pro and P38 (Figure 4 and [8]) provides additional evidence for the importance of this *RDR6*-dependent branch of silencing in antiviral defense. However, suppression of silencing by these viral proteins must involve more than inhibition of transitivity because they suppress hairpin transgene-induced silencing (Figure 4B and [39], [43], [47]), which does not require *RDR6* [8], [30]. One possibility is that viral suppressors also inhibit siRNA function by binding to siRNA duplexes [41], [42]. It will be interesting to determine whether inhibition of transitivity results from the dsRNA binding activity of the suppressors or reflects some additional activity.

Small RNA pathways have been shown to involve functional modules of specific gene family members, and modules can act alone or sequentially [4], [48], [49]. Thus, *DCL1* and *AGO1* are involved in the biogenesis and function of miRNAs, while *RDR2*, *DCL3*, and *AGO4* are involved in that of hc-siRNAs. *RDR6* and *DCL4* constitute a module that functions in the biogenesis of ta-siRNAs and works downstream of miRNA-directed cleavage. Our results suggest that *DCL2* and *RDR6* also constitute a module in the case of some substrates. These substrates differ from ones previously identified for the *DCL4/RDR6* module in that neither DCL4 nor DCL3 appears to process them in the absence of DCL2, perhaps due to unique structural features of the substrates or their localization in the cell. One possibility is that DCL2 might be required upstream of dsRNA production to recognize the substrates and/or recruit RDR6, after which DCL2 and other DCL enzymes produce siRNAs from the resultant dsRNA. Because the majority of secondary siRNAs in wild type plants are 21-nt for all three cases of silencing we examined, DCL4 appears to be overall the most active one of the enzymes on the *RDR6*-dependent dsRNA substrates produced by transgenes. Alternatively, 21-nt siRNAs might simply be the most stable. For all of three transgenes, however, 21-nt and 22-nt secondary siRNAs are equally abundant at the 3′ end of the GUS sequence, consistent with a specific requirement for *DCL2* early in the *RDR6*-dependent arm of transgene silencing. The processivity of DCL4 appears to be greater than that of DCL2 on these substrates because only 21-nt siRNAs accumulate from the middle region of the GUS sequence. This difference in processivity of the two enzymes might be at least partially responsible for the perception that DCL2 plays a purely subordinate role. The reduced accumulation of loop mRNA and primary siRNA in *dcl2* mutant plants (Figure 2B) suggests that DCL2 might also stabilize its substrates against non-silencing related nucleolytic degradation.

Because transitivity and sense transgene-induced silencing–like ta-siRNA biogenesis-require *RDR6*, DCL4 has been considered the likely DCL enzyme for siRNA production in sense transgene silencing. Moreover, siRNA production from a silenced sense transgene that was engineered to have a miRNA cleavage site was shown to utilize the *DCL4/RDR6* module after miRNA cleavage and not to require *DCL2* [8], providing support for the expected role of DCL4 in transitivity and sense transgene silencing. The present work, however, shows that *RDR6*-dependent production of siRNAs from transgenes that do not have a miRNA cleavage site differs from ta-siRNA biogenesis.

A number of studies have examined the *DCL* requirements of hairpin transgene induced silencing [24], [25], [36], [50]. The studies variously differ from one another and from our work with respect to structure of the hairpin transgene, dependence on a silencing signal, and whether the target of silencing is a transgene or an endogenous gene. Our study is the only one to date that has focused specifically on cell-autonomous silencing of transgenes and systematically distinguished between accumulation of primary and secondary siRNAs. Using this approach, we find that hairpin transgene-induced silencing occurs in *dcl2*, *dcl4*, and *dcl2 dcl4* mutants. The mechanism of target degradation varies, however, depending on which of the DCL enzymes are active. DCL4-dependent processing of the stem of the hairpin into primary siRNAs is the major pathway utilized in wild type and *dcl2* mutant plants. Inactivation of DCL4 promotes a shift to transitive silencing and the production of secondary siRNAs. The very high level of accumulation of secondary siRNAs in *dcl4* mutant plants transgenic for both the ΔGUS-SUG and 6b4 GUS loci (Figure 3B) suggests that under some conditions, secondary siRNA production itself is a major contributor to degradation of the targeted transcript, consistent with the observation that *AGO1* is not required for silencing of a GUS transgene by the ΔGUS-SUG locus [30]. When both DCL2 and DCL4 are inactive, silencing is again dependent on primary siRNAs, and processing of the stem of the hairpin by DCL3 becomes evident (Figures 2B and 3B). Our observation that DCL4 completely processes the 558-bp dsRNA stem of a hairpin construct into primary siRNAs, leaving only the loop portion (Figure 2A), suggests that the enzyme is highly processive on *RDR6*-independent dsRNA as well as on *RDR6*-dependent dsRNA (Figures 1B, 3B and [8]) and is consistent with the observation that *DCL4* is required when silencing depends entirely on production of primary siRNAs from a hairpin transgene [24].

The gene families involved in RNA silencing in plants have evolved to provide a large degree of functional diversity. It is striking that despite this potential for diversity, production of secondary siRNAs relies on *DCL2* in both sense and hairpin transgene silencing, which otherwise differ in their genetic requirements. The DCL enzymes and functional modules undoubtedly evolved to efficiently handle a wide variety of natural substrates–many of which have probably not yet been identified, and it is likely that the enzymes recognize signature structural features of their preferred substrates. The multiplicity of functional pairings is just beginning to be elucidated. For example, although RDR2/DCL3 is a well established module involved in hc-siRNA biogenesis, DCL3 activity without RDR2 and the pairing of RDR2 with DCL4 has recently been proposed for a particular case of hairpin transgene targeting of an endogenous gene [48]. The diversity and great versatility of small RNA pathways in plants is perfectly exemplified by how well prepared plants turned out to be to defend themselves against the recent evolution of genetic engineers.

## Materials and Methods

### Transgenic and mutant *Arabidopsis* lines

All lines are in the Columbia (Col-0) ecotype. The following transgenic or mutant lines were described previously: L1 [33], 306-1 and 6b4/306 [30], P38 (CP) [40], P1/HC-Pro [51], *sgs2-1 (rdr6)* [52], *dcl1-7* and *dcl1-8* [53], *dcl2-1* (SALK_064627) and *dcl4-2* [23]. The *dcl4-10* mutation is a previously unpublished mutation that arose in an EMS mutagenesis [16]. It is a single mucleotide mutation that produces a glycine to arginine change at amino acid 1403, which is located in the RNAse III domain of DCL4 (data not shown).

### PCR genotyping for mutant and transgene loci

The T-DNA primer LBa1 (tgg ttc acg tag tgg gcc atc g) was used with primers DCL2p5 (ttg gat tgc atg cac aca tt) and DCL2p6 (ctc aga aat aaa gat aac agt aag caa at) for *dcl2-1* genotyping. Primer DCL2p5 together with DCL2p6 amplifies a 400-bp product from the wild type locus, while DCL2p5 together with LBa1 amplifies a 600-bp product from the *dcl2-1* locus. Thus, a PCR reaction with all three primers will amplify only the 600-bp product in the case of homozygous *dcl2-1*. Genotyping for homozygous *dcl4-2* was performed as described previously [23]. Homozygous *dcl4-10* plants containing the L1 GUS transgene were identified by their distinctive leaf phenotype, which is similar to that of the *dcl4-2* mutant [23]. Primers L1-306-6b4-F (ttg ggg ttt cta cag gac gga c) and L1-306-6b4-R (cta tcc ttc gca aga ccc ttc c) were used in combination with GUS staining to screen for the presence of different GUS loci. A 250-bp fragment is obtained with the 306-1 locus, a 188 bp fragment with the L1 locus and a 127-bp fragment with the 6b4 locus, due to differences in the 5′ upstream sequences of the GUS constructs. Homozygous *dcl1-7* and *dcl1-8* plants containing the L1 GUS transgene were identified by their distinctive recessive phenotypic defects [53]. Primers 2911F7 (gca ggg ata ctt gaa cat ggc c) and 2911R8 (gtt aac aac cta tgc cac gc) were used for *sgs2-1* genotyping: PCR followed by digestion with BstNI yields three fragments (sizes 250, 200, and 150 bp) from the wild type locus, but only two fragments (200 and 400 bp) from the *sgs2-1* locus. P38-derived primers P38-F (cgc cca atg ggc gat aaa g) and P38-R (cgt ctc ggt cga atg cca gag c) were used to confirm the presence of the P38 transgene in combination with phenotypic and phosphoinothricin selection.

RNA isolation, gel blot analysis, and nuclear run-off transcription. RNA was isolated from a mixture of representative aerial tissues of flowering plants. For most experiments, tissues from about ten plants of a given genotype were pooled for RNA isolation. For each genotype, at least two independent RNA preparations were made from separate plants or pools of plants and electrophoresed in neighboring lanes on RNA gels. Total RNA isolation and gel blot analysis of high and low molecular weight RNA were performed as described previously [51], [54]. Nuclear run-off transcription analysis was performed as described previously [55] except that nuclei were isolated from aerial tissues of flowering plants.

The minimal sequence (taa tac gac tca cta tag gg) of the T7 promoter was incorporated by PCR into the 3′ or 5′ ends of DNA templates to make RNA probes of antisense polarity to detect mRNAs or of sense polarity to detect antisense siRNAs, respectively. [α-^32^P]UTP-labeled RNA probes were transcribed in vitro using an Ambion MAXIscript kit with T7 polymerase and hybridized to mRNA blots at 68°C in Ambion ULTRAhyb buffer, or to siRNA blots at 42°C in Ambion ULTRAhyb-oligo buffer. DNA probes were labeled using an Ambion DECAprime II kit and hybridized to mRNA blots in Ambion ULTRAhyb buffer at 42°C. The coding sequence coordinates of the probes for viral suppressors of silencing were: P38, nucleotides 42 to 472; HC-Pro, entire coding sequence. The siR255 probe was prepared by end-labeling the complementary DNA oligonucleotide with [α-^32^P]ATP using the StarFire™ Oligo Labelling System (Integrated DNA Technologies) as described previously [56]. The probe was hybridized to small RNA blots at 42°C in Ambion ULTRAhyb-oligo buffer.
